# A novel role of the natriuretic peptide/cGMP/cGKI pathway in melanoma cells

**DOI:** 10.1186/2050-6511-14-S1-P19

**Published:** 2013-08-29

**Authors:** Sandeep Dhayade, Susanne Feil, Christoph Griessinger, Manfred Kneilling, Birgit Schittek, Robert Feil

**Affiliations:** 1 Interfakultäres Institut für Biochemie, University of Tübingen, Tübingen, Germany; 2 Department of Preclinical Imaging and Radiopharmacy, Laboratory for Preclinical Imaging and Imaging Technology of the Werner Siemens-Foundation, University of Tübingen, Tübingen, Germany; 3 Department of Dermatology, University of Tübingen, Tübingen, Germany

## Background

The cGMP/cGMP-dependent protein kinase type I (cGKI) signaling pathway is activated by nitric oxide (NO), natriuretic peptides (ANP, BNP & CNP), and cGMP-elevating drugs. It regulates important physiological functions such as platelet aggregation, smooth muscle tonus, and cell growth and survival. Recent reports indicate that cGMP might also play a role in tumorigenesis. In the present study we found that cGKI is expressed in melanoma cells of murine and human origin.

## Results

Treatment of intact mouse B16 melanoma cells with the membrane-permeable cGMP analog 8-Br-cGMP induced phosphorylation of the cGKI substrates, vasodilator-stimulated phosphoprotein and phosphodiesterase 5. ANP and CNP, ligands of the membrane-bound guanylyl cyclase GC-A and GC-B, respectively, activated the endogenous cGMP/cGKI pathway. CNP-induced cGMP signals were detected in cell extracts by ELISA and in living cells by a FRET-based cGMP sensor [1]. DEA/NO, which stimulates NO-sensitive soluble guanylyl cyclase, did not increase cGMP signaling in B16 cells. Interestingly, activation of cGMP/cGKI signal transduction was associated with an increase in ERK1/2 and p38 phosphorylation, growth and migration of B16 melanoma cells. Similar results were obtained with WM1205 human melanoma cells.

## Conclusion

We have identified a natriuretic peptide/cGMP/cGKI pathway in melanoma cells, which stimulates tumor cell growth and migration in vitro. Pharmacologic inhibition of cGMP signaling may offer a promising strategy for the treatment of melanoma.
